# Evaluation of “*Caterina assay*”: An Alternative Tool to the Commercialized Kits Used for Severe Acute Respiratory Syndrome Coronavirus 2 (SARS-CoV-2) Identification

**DOI:** 10.3390/pathogens10030325

**Published:** 2021-03-10

**Authors:** Germano Orrù, Alessandra Scano, Sara Fais, Miriam Loddo, Mauro Giovanni Carta, Giorgio Carlo Steri, Simonetta Santus, Riccardo Cappai, Maria Laura Ferrando, Ferdinando Coghe

**Affiliations:** 1 Department of Surgical Sciences, Molecular Biology Service (MBS), University of Cagliari, 09124 Cagliari, Italy; alessandrascano@libero.it (A.S.); sarafais79@gmail.com (S.F.); laura_ferrando@yahoo.it (M.L.F.); 2 Dipartimento Servizi di Diagnosi e Cura, Azienda Ospedaliero-Universitaria di Cagliari (A.O.U.), University of Cagliari, 09024 Cagliari, Italy; m.loddo@aoucagliari.it (M.L.); r.cappai@aoucagliari.it (R.C.); fcoghe@aoucagliari.it (F.C.); 3 Department of Medical Sciences and Public Health, University of Cagliari, 09124 Cagliari, Italy; mgcarta@tiscali.it; 4 Azienda per la Tutela della Salute (ATS), Aree Socio-Sanitarie Locali (ASSL) of Cagliari, 09131 Cagliari, Italy; dir.generale@atssardegna.it (G.C.S.); simonetta.santus@atssardegna.it (S.S.)

**Keywords:** molecular diagnostic of SARS-CoV-2, COVID-19 detection, SYBR^®^Green real-time PCR, N protein, melting curve analysis, DNA folding

## Abstract

Here we describe the first molecular test developed in the early stage of the pandemic to diagnose the first cases of severe acute respiratory syndrome coronavirus 2 (SARS-CoV-2) infection in Sardinian patients in February–March 2020, when diagnostic certified methodology had not yet been adopted by clinical microbiology laboratories. The “*Caterina assay*” is a SYBR^®^Green real-time reverse-transcription polymerase chain reaction (rRT-PCR), designed to detect the nucleocapsid phosphoprotein (N) gene that exhibits high discriminative variation RNA sequence among bat and human coronaviruses. The molecular method was applied to detect SARS-CoV-2 in nasal swabs collected from 2110 suspected cases. The study article describes the first molecular test developed in the early stage of the declared pandemic to identify the coronavirus disease 2019 (COVID-19) in Sardinian patients in February–March 2020, when a diagnostic certified methodology had not yet been adopted by clinical microbiology laboratories. The assay presented high specificity and sensitivity (with a detection limit ≥50 viral genomes/μL). No false-positives were detected, as confirmed by the comparison with two certified commercial kits. Although other validated molecular methods are currently in use, the *Caterina assay* still represents a valid and low-cost detection procedure that could be applied in countries with limited economic resources.

## 1. Introduction

The current pandemic of coronavirus disease 2019 (COVID-19), caused by severe acute respiratory syndrome coronavirus 2 (SARS-CoV-2) [1], poses serious problems in different fields of medical practice [2,3]. COVID-19 respiratory infection may vary from very mild to severe symptoms, even death, and presents clinical features that render it difficult to diagnose it from other viral respiratory diseases [4,5]. SARS-CoV-2 contains a single-stranded RNA as genome (~30 kb) and is a zoonotic agent likely originated from bats and genetically related to other human beta-coronaviruses, SARS-CoV and Middle East respiratory syndrome–related coronavirus (MERS-CoV), albeit with substantial differences in virulence and pathogenicity profile [6].

In December 2020 over 78 million cases and about 1.72 million associated COVID-19 deaths had been reached (based on World Health Organizations (WHO) weekly epidemiological update) [7] and still new resolutive approaches in the diagnosis and therapy for COVID-19 are needed. Diagnostic laboratory tests to detect the presence of COVID-19 are pivotal tools for epidemiological assessment of the declared pandemic [8,9,10].

The methods for the specific detection of SARS-CoV-2 were based on real-time reverse-transcription polymerase chain reaction (rRT-PCR), most of them to reveal the genetic material through the use of multiple primers and hydrolysis probes designed on RNA sequences of one or more reference (i.e., TaqMan probe) [11]. Currently, the main gene targets used in COVID-19 diagnosis by main health organizations are related to N (nucleocapsid phosphoprotein, N1, N2 and N3 amplicons, Centers for Disease Control and Prevention (CDC) [12] and N2 amplicon WHO [13]), E (envelope protein, E amplicon, WHO [13,14]) and ORF-1ab (RdRP gene, ab polyprotein) RNA region [13,15,16,17]. 

In 2020, an evolution of the detection methodology took part and can be listed in three phases: (i) in January and February 2020, setup of “homemade assays”; (ii) in March and April, appeared the first commercial kits; (iii) from May until today, detection methods were set by different governments guidelines. These mentioned virtual periods had different approaches in terms of accuracy due to available kits, analytical procedures, sampling, detection and results dissemination.

Our study falls in the first period in January 2020 [18] and describes the setup of a low-cost “homemade assay” based on a double-stranded DNA binding dye (SYBR^®^Green) rRT-PCR, [18] used in our Azienda Ospedaliero-Universitaria (AOU), Cagliari (Italy) for diagnosing the first suspected cases of COVID-19. The method was subsequently validated by SYBR^®^Green melting curve analysis and PCR amplicon sequencing. This molecular assay, named *Caterina assay*, allowed the detection of the first COVID-19 patient (“patient zero”) in the Sardinia region and remained in use for the first few months following the start of the pandemic, helping to contain the spread of the infection. 

Aim of the study: to evaluate the validity and reliability of the *Caterina assay* for COVID-19 detection.

## 2. Materials and Methods

### 2.1. SARS-CoV-2 rRT-PCR In Silico Design

**Figure 1:**
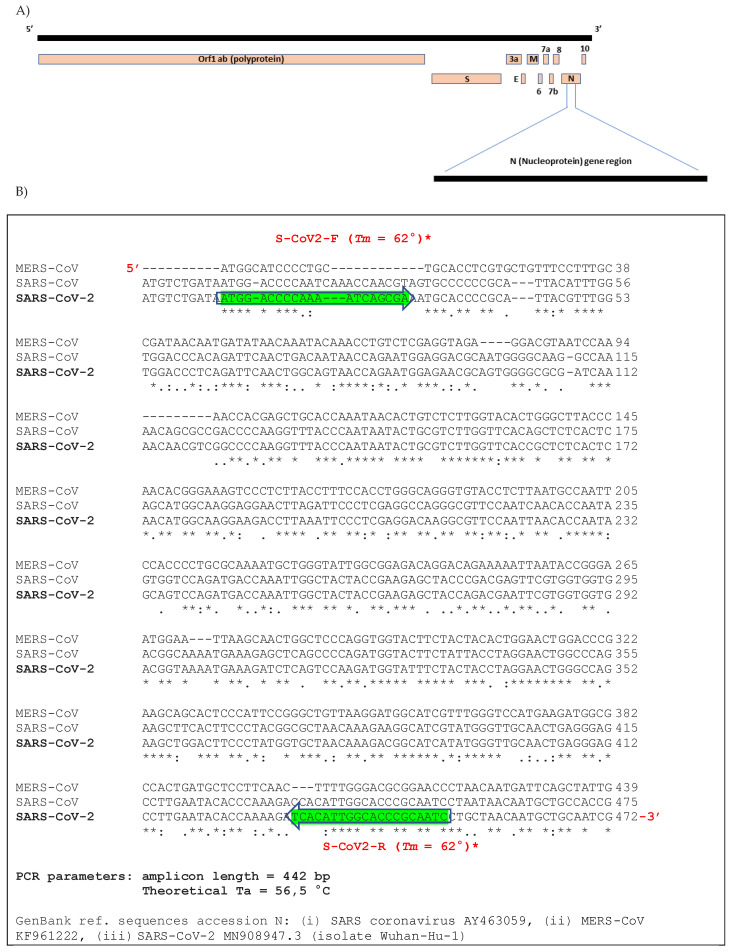
(**A**) Map of severe acute respiratory syndrome coronavirus 2 (SARS-CoV-2) genome showing the position of primers designed on the N-terminal region of N protein (N1). Several open reading frames (ORFs) include accessory genes (ORF 1a, 1b, 3a, 6, 7a, 7b, 8, and 10) and structural elements such as S: spike protein gene, E: envelope protein gene, M: membrane protein gene, N: nucleocapsid protein gene. ORF-1b encodes RNA-dependent RNA polymerase (RdRp) gene (**B**) Partial region of N1 gene sequence of SARS-CoV-2 aligned with the other two most related outbreak human beta-coronaviruses (SARS and MERS), and position of the oligonucleotides with the respective Tm parameters, by the Marmur–Doty formula* used in this work (T_m_ = 2 (A + T) + 4 (C + G) − 7).

Forward and reverse primers were designed on the genome sequence of SARS-CoV-2 Wu-Hu-1 accession number MN908947.3 in position 28284–28303 nucleotides (nt) (S-CoV2-F 5′-ATGGACCCCAAAATCAGCGA-3, 11–30 nt within N gene) and 28705–28724 nt (S-CoV2-R 5′-GATTGCGGGTGCCAATGTGA-3, 432–451 nt within N gene) of the N-terminal region of N gene (N1) to amply a PCR product of 451 bp (Figure 1). Possible oligos dimers formation and/or self-complementarity and theoretical melting temperatures of primers (Tm) were calculated using Oligo program version 6 (MedProbe, Oslo, Norway). Oligos hybridization were set with the following parameters: monovalent cation concentration of 0.05 mol/L, Mg^2+^ at 0.002 mol/L, a concentration of probe and target of 100 mM, initial hybridization temperature of 37 °C [17].

**Figure 2:**
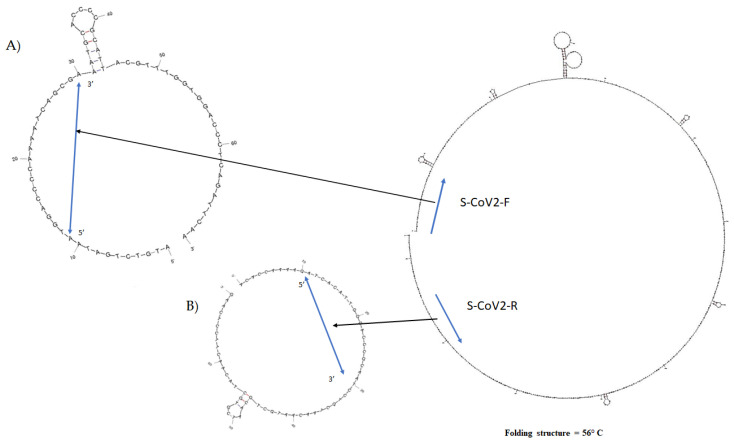
Example for primer design following the stem-loop architecture in the amplicon N gene region, in the same positions described in Figure 1. Secondary structures positioned on (**A**) S-CoV2 forward primer and (**B**) S-CoV2 reverse primer sequence. This *mFold* analysis showed the absence of D-loops that could interfere with the PCR sensitivity [19].

*mFold* program (http://www.unafold.org/DNA_form.php accessed on 20 January 2020) to predict D-loop within the N gene target was used as already published [19,20] (Figure 2). Briefly, for the selected target sequence, folding conditions were Na^+^ 0.05 mol/L; Mg^++^ 0.002 mol/L. The hybridization temperature corresponded to the Sanger PCR annealing temperature (Ta), as indicated in Figure 2.

### 2.2. Positive Control 

Initially, an RNA sample extracted from a nasal swab of a patient with confirmed clinical diagnosis of COVID-19 was utilized as positive control. Later, Opitrol NAT SARS-CoV-2 (http://www.serobac.at/downloads/Serobac-IFU%20Optitrol%20NAT%20SARS-CoV-2.pdf (accessed on 7 March 2021), DiaMex GmbH, Siemensstr. 38, 69123 Heidelberg, Germany) was utilized as positive control to monitor the performance of our in vitro assay. NAT SARS-CoV-2 contained an inactivated cell culture supernatant with intact SARS-CoV-2 with viral titer ±1 × 10^7^ corresponding to 1 × 10^7^ viral genomes/mL. 

### 2.3. RNA Extraction

RNA was extracted by using a QIAamp^®^ viral RNA Mini Kit (QIAGEN GmbH, Hilden, Germany) following the manufacturer’s instructions. The same extraction was used for SARS-CoV-2 cell suspension extraction (positive control) as well as for the clinical samples (swabs) used in the routine work. Before the PCR analysis, all extracts were analyzed for total RNA concentration and purity by using NanoDrop™ OneC Spectrophotometer (Fisher Scientific Pte Ltd, 8 Pandan Crescent LL4, Singapore).

### 2.4. RNA Extraction Internal Controls

A set of oligonucleotides, designed on human β-actin gene (GenBank accession N. M10277), was used as internal control to assess the correct RNA extraction procedure. The oligos β-forward 5′-GGCGTGATGGTGGGC-3′ and β-reverse 5′-GTCATCTTCTCGCGGTTG-3′ were used in the real-time PCR reaction, by using the same conditions of SARS-CoV-2 PCR, and the amplicon length was 236 bp.

### 2.5. Real-Time rt-PCR Quantitative Conditions 

The SYBR^®^Green Real-time PCR reaction was carried out with TaqPathTM 1-Step RRT-PCR MasterMix (Life Technologies, Corp in Carlsbad, Carlsbad, CA, USA). Briefly, 20 µL final volume contained 5 µL master mix solution (4×), 1 µL SYBR^®^Green (Sigma–Aldrich, St. Louis, MO, USA) solution 1/1000, 1 µL of 100 µM solution of each primer, 5 µL RNA extract and 7 µL of DNase RNase free water. Additionally, heat-labile uracil–DNA–glycosylase (UDG, Roche Molecular Biochemicals) and 2 L of RNA extract was used. The PCR profile, conducted by using a CFX 96 apparatus (Bio-Rad laboratories USA), was as follows: (i) an initial uracil–DNA–glycosylase (UNG) incubation at 25 °C for 2 min, (ii) RT incubation at 50 °C for 15 min and (iii) 40 cycles of 3 s at 95 °C, 30 s at 60 °C and 2 s at 83 °C, (iv) final melting curve was performed for 50 to 95 °C with a transition rate of 5 °C/s. Fluorescence was detected at the end of the 83 °C segment (avoiding a specific fluorescence due to primer dimers and in continuous mode during melting curve process). To ensure the absence of potential secondary PCR products, amplicons were analyzed by 0.8% agarose gel electrophoresis.

### 2.6. cDNA Sequencing

To confirm that the obtained PCR product amplified the expected N gene region of SARS-CoV-2, a sequencing reaction (Sanger reaction) was performed using the Big-Dye-chemistry Kit Perkin–Elmer Applied Biosystems, Foster City, CA, USA) and the same oligos utilized in rRT-PCR as sequencing primers. The results were edited and evaluated with Chromas chromatogram file editor (Technelysium, Queensland, Australia) and analyzed by the Basic Local Alignment Search Tool program (Blastn, http://www.ncbi.nlm.nih.gov/BLAST accessed on 20 March 2020).

### 2.7. Clinical Samples 

There were 2110 nasopharyngeal swabs tested from patients recorded as suspected cases (with clinical signs of COVID-19), or contact (close contact with an individual with COVID-19). The group of tested patients was composed of 58% women, aged (20–99) median 59 and 42% men, aged (20–80) median 55. All samples were recruited from January to March 2020. Nasal swab sampling was performed using FLOQSwabs^®^ (Copan Italia S.p.A., Brescia, Italy), collected into a tube containing MEM transport media and stored at 4 °C until RNA extraction was performed on the same day of the sampling. In each run a no template control, negative extraction control, and SARS-CoV-2-positive control were included as previously described. Samples were considered negative when the SARS-CoV-2 target had a cycle threshold (Ct) >40.

### 2.8. Statistical Analysis

The experiment was executed in triplicate; the standard deviation (SD) of the threshold cycles Ct for each sample were comprised between ±0.8 Ct. The correlation coefficient (R2) for the standard curve was comprised of 0.95 to 0.97, and the different PCR efficiency was comprised between 97% and 98%. The accuracy levels of this analytical procedure were measured by Cohen’s kappa coefficient (k) [18] by using the “Kappa as a Measure of Concordance in Categorical Sorting/VassarStats” program, available online http://vassarstats.net/kappa.html (accessed on 21 December 2020), all with 95% confidence intervals.

## 3. Results

### 3.1. Primer Design

PCR primers were designed on the nucleotide sequence of SARS-CoV-2 Wu-Hu-1 accession number MN908947.3 (now deposited in PubMed https://www.ncbi.nlm.nih.gov/nuccore/MN908947 accessed on 17 January 2020), which was posted for the first time in the open access Virological website (http:// virological.org/) on 11 January 2020 and now also available in Global Initiative on Sharing lll Influenza Data (GISAID, https://www.gisaid.org/ accessed on 20 January 2020) (Figure 1A). The whole genome sequence was aligned with available sequences of bat and human-associated beta-coronavirus: Bat-SARS-CoV (FJ588686) HCoV-HKU1 (AY597011, KF430201), HCoV-NL63 (KF530114, MK334043), HCoV-OC43 (MK303625, KF530099, MN306042), MERS-CoV (KF961222, KT006149, MG923473, MF593268), SARS-coronavirus [DQ640652, AY274119, MK062184 (Urbani strain), GU553364, AY463059. Among them, the N gene of SARS-CoV-2 (28274–29533 nt) showed the most discriminative region between the aligned sequences (data not shown). In details, a multiple alignment of the N1 gene revealed a high sequence variability between the analyzed beta-coronaviruses (Figure 1B). To avoid problems of steric effects between PCR primers and gene target, an accurate cDNA fold evaluation was performed by the *mFold* web program as already published [19] (Figure 2). The selected oligos were designed in a region without displacement loop (D-loop) and characterized by a low Gibbs free energy (∆G^0^) value [21] (Figure 2).

### 3.2. SYBR^®^Green rRT-PCR Melting Curve Analysis

**Figure 3:**
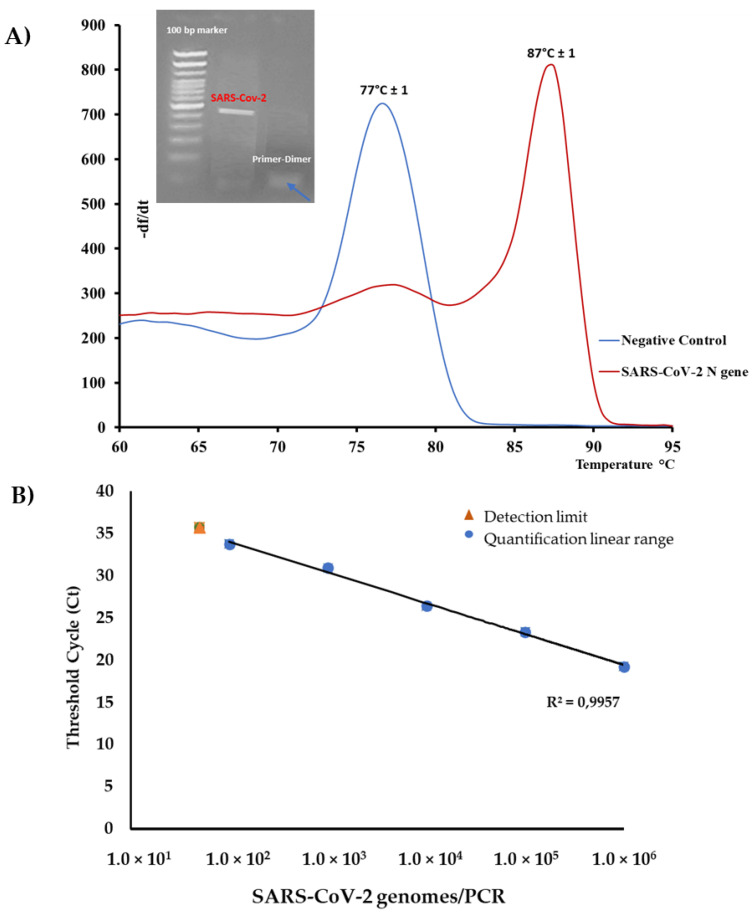
(**A**) Real-time PCR melting curve and agarose gel profiles of a positive nasopharyngeal swab and respective negative control. The melting peak at 77 °C ± 1 °C in negative control as well as the small band in agarose gel, represents a primer dimer product. (**B**) rRT-PCR standard curve obtained with serial dilution of SARS-CoV-2 inactivated virions. The linear range of quantitation was 10^2^–10^6^ viral genomes/uL, while the detection limit was observed to 50 viral genomes/uL.

The melting curve analysis of the N gene amplicons revealed two different melting peaks spaced by approximately 10 °C: the first peak, with a Tm of 77 ± 1 °C, represented possible primer-dimers that were relievable in the negative control/sample (around 40 bp). The second peak with a Tm of 87 ± 1 °C corresponded to the SARS-CoV-2 positive sample (451 bp). The same result was verified by agarose gel electrophoresis analysis, in which the two peaks in fluorescence corresponded to two PCR bands presenting different sizes (Figure 3A).

**Figure 4:**
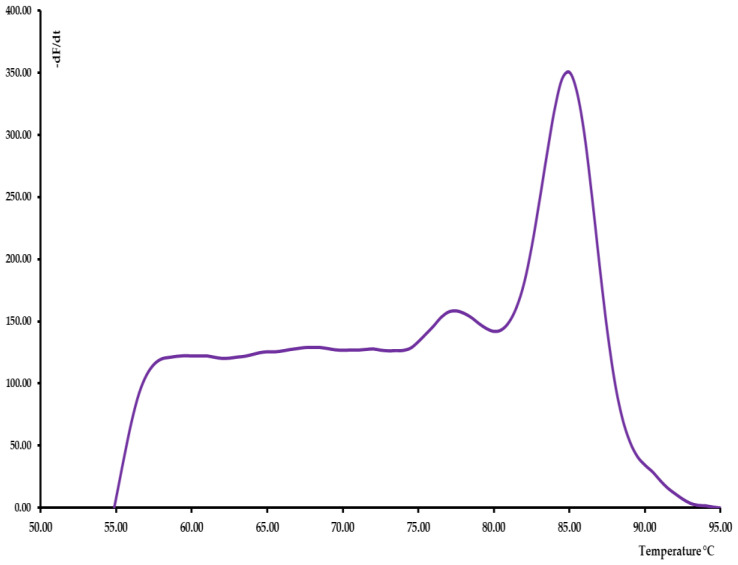
rRT-PCR melting curve for an extraction control of a positive nasopharyngeal swab for human β-actin gene. An 85 °C melting peak indicated a correct RNA extraction procedure, and all samples were considered valid with a Ct ≤ 30.

rRT-PCR melting curve analysis was also performed for human β-actin gene that was used as molecular target control for RNA extraction (Figure 4). A positive reaction was confirmed by a melting peak of 85 °C Tm and a rRT-PCR Cycle threshold (Ct) limit ≤31. 

### 3.3. Sensitivity of the Method

Inactivated Opitrol NAT SARS-CoV-2 was utilized to evaluate the sensitivity of the method. Opitrol NAT SARS-CoV-2 was provided with a viral titer ±1 × 10^7^ corresponding to 1 × 10^7^ viral genomes/mL. The calibration curve for the genomic copy number versus Ct value was obtained from 10-fold serial dilutions of the viral suspension, ranging from 1 × 10^6^ to 50 viral genomes/μL extract (with Ct = 35.75), and served as a standard quantification curve to evaluate the sensitivity of the rRT-PCR reaction. 

A wide linear range of quantitation was obtained (from 10^6^ to 10^2^ viral genomes/uL, 7 ten-fold dilutions). The standard curves showed a correlation regression coefficient R^2^ = 0.99 (Figure 3B).

### 3.4. SARS-CoV-2 N Protein Identification by Sequencing

**Figure 5:**
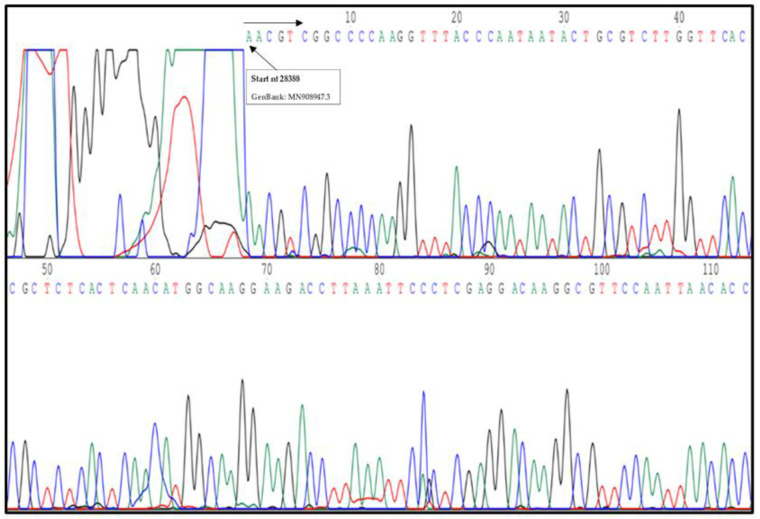
Chromatogram of DNA sequencing of N gene SARS-CoV-2 PCR amplicon fragment. The arrows indicate the first nt of N gene of reference genome SARS-CoV-2 Wu-Hu-1 deposited on GeneBank.

In order to confirm the specificity of the “*Caterina assay*” as a diagnostic tool for SARS-CoV-2 detection, 10 qPCR amplicons from clinical specimens were sequenced by the Sanger method [19]. Both rRT-PCR primers were used in the Sanger sequencing reaction to generate a sequence of 406 bp (Figure 5). Nucleotide sequence showed 100% of homology with the N gene of the reference genome SARS-CoV-2 Wu-Hu-1, as previously evaluated in silico analysis. In addition, we also verified by sequencing the amplicons of 50 samples (from February to April 2020). The first sequence of the N1 gene was deposited in the nucleotide database GenBank with accession MT187977.1.

### 3.5. Detection of SARS-CoV-2 on Clinical Samples from a Court of Sardinian Patients

The recruitment of clinical samples was organized within a monitor COVID-19 program established by the Sardinian regional government through the “Azienda Tutela della Salute” (ATS). 

In total, there were 2110 oropharyngeal swabs collected from public hospital patients with suspected COVID-19 infection, composed of 1230 females and 880 males. Samples swabs were collected for a period of 60 days starting from 3 February 2020. The clinical samples were collected from (a) suspected patients for clinical signs (*n* = 648) and asymptomatic patients (*n* = 1462). Among 2110 samples analyzed with the “*Caterina assay*”, 181 samples resulted SARS-CoV-2 positive (8.5%) and 1900 negative (91.5%). Fifty positive samples were further sequenced to confirm the expected sequence of SARS-CoV-2 N1 gene (data not shown). No mutation was detected with sequenced qPCR amplicons, confirming the relative stability of the N1 gene of the SARS-CoV-2 strains isolated from Sardinian patients.

**Figure 6:**
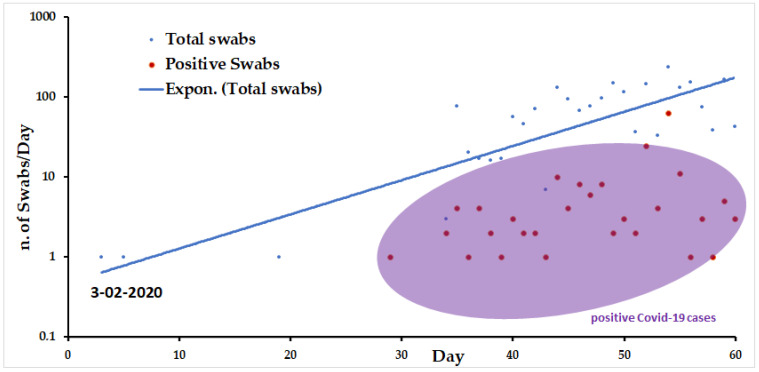
Number of swabs/days analyzed by *Caterina assay* during the initial epidemic period in Sardinia. Blue dots indicate negative swab samples while red dots indicate positive swabs and the eclipse purple visualizes the area with the highest numbers of positive swabs plotted in the graph. An increase of positive samples was observed from 29 February 2020 (“patient zero”).

Figure 6 shows the number of total swabs analyzed and the relative positive samples/day observed during the initial pandemic period in Sardinia. The first positive sample was revealed 29 February 2020 (30 days from the set-up of the application of the “*Caterina assay*”). Subsequently, the positive samples began to increase over two months, as a consequence of an increase in the number of swabs per day more than the number of cases itself, with a maximum number of positive swabs (*n* = 63) detected. The percentage of positive samples between genders was 9.1% and 7.9%, females and males respectively.

### 3.6. PCR Validation Method: Comparison of Commercial Kit and the Homemade Procedure

After the first months of the epidemic, as soon as commercial validated kits were available, SARS-CoV-2 diagnosis was carried out in conjunction with the use of the *Caterina assay* with two commercial kits based on Taq-Man probe rRT-PCR, providing the utilization of three different tests to analyze a single clinical sample. Both commercial kits were recommended by WHO for in vitro diagnostics of SARS-CoV-2 (https://www.who.int/diagnostics_laboratory/200708_eul_sars_cov2_product_list.pdf?ua=1 accessed on 27 December 2020): (1) Genesis-coronavirus (Primer Design, York-House, School lane, UK) and (2) DA An Gene Co., Ltd. (Sun Yat-Sen University, Guangzhou, Guangdong, China). 

There were 110 samples recruited from hospitalized patients with a clinical and laboratory diagnosis for COVID-19, and 100 samples from asymptomatic subjects, which were used to calculate the concordance between molecular tests. Inter-rater reliability was calculated by Cohen’s kappa coefficient by GraphPad online calculator, (https://www.graphpad.com/quickcalcs/kappa1/ accessed on 5 January 2021). The *Caterina assay* showed an optimal intra-rater reliability with both diagnostic tests, used as gold standard in this experiment. The K coefficient was ranged from 0.97 (95% CI, 0.939–1.0) to 0.99 (95% CI, 0.972–1.0) for COVID-19 Genesig Real-Time PCR assay^TM^ and DA An Gene (2019-nCoV) RNA^TM^ respectively (Table 1).

## 4. Discussion

With the WHO declaration of the COVID-19 pandemic, countries were faced with this novel public health emergency [22]. More accurate and sensitive diagnostic methods are needed to monitor new cases and the progress of the pandemic.

SARS-CoV-2 diagnostic is constantly evolving and the novel advanced molecular techniques, including multiplex rRT-PCR [23,24], CRISPR–Cas12 [17] and loop mediated isothermal amplification (LAMP) [25], promise to increase the analytical sensitivity and testing capacity.

Our study describes a low-cost and simple diagnostic method used in Sardinia (Italy) to report cases and assess the declared pandemic when commercial kits and information on the genome sequences of the new SARS-CoV-2 were not yet available. The “*Caterina assay*” analytical procedure was designed according the first SARS-CoV-2 genome sequenced by Zhang et al. [26] and the available genomes of other SARS-associated beta-coronaviruses in human and other animal reservoirs (Figure 1). It has been in use in our medical service laboratory from January 2020 to the beginning of April 2020 and enabled a fast and reliable diagnosis of the first COVID-19 cases (Figure 6). A carful study of the stem–loop architecture in the N1 gene amplicon permitted to identify a looped-out region from the primer binding site where the oligos could perfectly anneal to the template, increasing the sensibility of the detection method (Figure 2) [21,27]. Indeed, the reaction presented a high sensitivity (detection limit to 50 viral genomes/μL, Ct = 35.75) (Figure 3), which resulted to be higher compared to other SYBR^®^Green-based RT-PCR methods for SARS-CoV-2 detection (10^3^ copies/µL) [28]. Specificity of detection of SARS-CoV-2 was finally confirmed by the sequencing of 50 RT-PCR amplicons coming from clinal specimens (Figure 5). 

Among the “gold standards” developed for SARS-CoV-2 detection, the structural viral protein E and N genes [12,13,17], and non-structural protein RdRp [13,17] were the most often chosen. The N protein especially was selected in first place during this study and by other authors due to the high sequence variation in comparison with the sequences of other related human and bat beta-coronaviruses (Figure 1) [12,13]. 

On the other hand, a comparison between the choice of the most specific and conserved target gene for diagnostic is often difficult, as the RT-PCR performance depends on which region primers were designed within a gene [27,29]. For instance, during the analytical comparison between primers/probe sets, RdRp and E genes were more sensitive than N genes [13,27]. However, Chu et al. found that the N gene assay was approximately 10 times more sensitive than the RdRp (ORF-1b) gene assay in detecting positive clinical specimens from two patients via rRT-PCR [30]. It has been reported that the N gene that encoded an internal N nucleoprotein protein was one of the most abundant proteins and was highly immunogenic and less prone to genetic changes [31,32]. However, recent study showed that some structural proteins, like the E protein, have low mutation rates across the residue sequence while other viral components, such as the Spike (S) [33] or the N protein showed higher degrees of variability [34]. 

**Figure 7:**
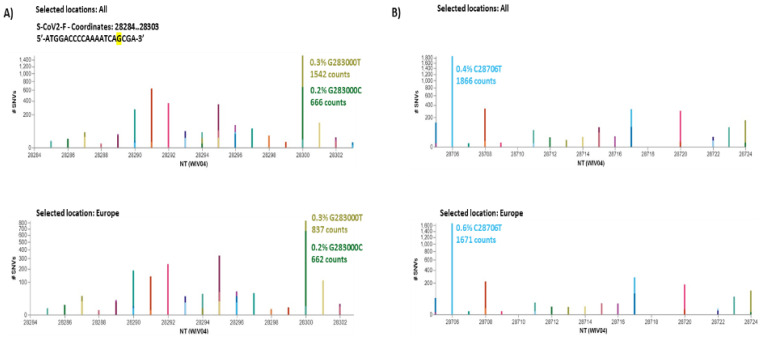
Frequency of mutations (single-nucleotide variations: SNVs (bold underline) performed with COVID-19 CG revealed in the counts of isolates sequenced collected worldwide (upper panel) and in Europe only (lower panel) of primers (**A**) S-CoV2-F (5′-ATGGACCCCAAAATCA**G**CGA-3′) 28284–28303 nt in which **G**283000**T** SNVs was present in 1542 sequence (0.4%) counts among the 443.126 sequences selected from isolates globally reported till to 12 February 2021 and (**B**) Reverse primers used in this study and 28705–28724 nt (S-CoV2-R 5′-GATTGCGGGTGCCAATGTGA-3′), 432–451 nt) in which **C28706T** was present in 1866 counts (0.4%) among the 443.126 sequences selected from isolates globally reported till to 12/02/2021.

Mutations present in the proximity of three-end oligo could affect primer sensitivity interfering with DNA elongation mediated by the activity of 5′-3′ exonuclease activity of the DNA polymerase. For this, we have evaluated through the COVID-19 CG browser interface (https://covidcg.org accessed on 18 February 2021), the frequency of mutations (single-nucleotide variations: SNVs) present in the primers sequence of the N1 protein [35]. Forward primer S-CoV2-F contained one SVN with a higher frequency of 0.4% in position 283,000 (5′-ATGGACCCCAAAATCA**G**CGA**-3′ G**283000**T** present in 1542 sequence counts among the 443.126 sequences selected from isolates globally collected) (Figure 7). This mutation could potentially impact the sensitivity of the primer. To overcome this potential issue, the use of a degenerative forward primer might be suggested.

**Figure 8:**
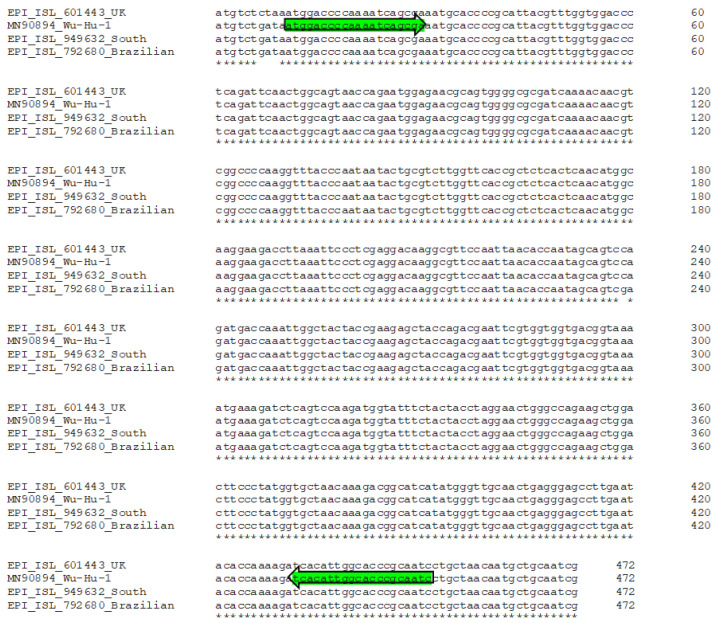
Multiple alignment of N1 region protein sequence alignments of SARS-CoV-2 Wu-Hu-1 isolate and emerging variants linked to potential higher transmissibility and infectivity (* indicate nt conservation among strains) [30]. UK lineage B.1.1.7 (hCoV-19/England/MILK-9E05B3/2020, GISAID ID: EPI_ISL_601443), Brazilian linage P.1 (hCoV-19/Japan/IC-0561/2021, GISAID ID: EPI_ISL_792680) and South African lineage B.1.351 (hCoV-19/South Africa/UFS-VIRO-NGS-163/2020, GISAID ID: EPI_ISL_949632) and reference sequence of SARS-CoV-2 Wu-Hu-1 accession number MN908947.3.

We also performed a multiple alignment of SARS-CoV-2 isolates considered emerging variants with a higher transmissibility and infectivity [36]. As shown in Figure 8, the primers of our diagnostic method were also able to detect the new variants and no mismatches was found in the annealing sequence of the primers [31,32].

Recently, a few papers have described alternative low-cost diagnostic methods for SARS-CoV-2 based on SYBR^®^Green chemistry. For instance, Topan et al. selected another conserved gene encoding the viral Membrane protein (M) to be detected by TaqMan probe or SYBR^®^Green. However, SYBR^®^Green qPCR resulted in being an adaptation of pre-existent TaqMan qPCR protocol, whose reaction could result in a lower amplification efficiency losing analytical sensitivity. 

Diagnostic performance of the *Caterina assay* has been benchmarked with official gold standards and is now available for diagnostic routine but not in the early epidemic phase. We accurately assessed the performance of the rRT-PCR reaction by testing previous patient samples tested with the *Caterina assay* using two gold standard kits (Table 1). Our analytical procedure still represents a robust and low-cost methodology that could be applied in particular settings. For example, the calculation of the burden of the coronavirus detection will be valuable: the medium of costs of a commercial diagnostic PCR kit (without extraction) is approximately 17 euros/sample, whereas the *Caterina assay* is about 3 euros/sample. The use of the *Caterina assay* may be valuable in low-resource countries where a low health income could be a critical condition during the governance of the COVID-19 pandemic. For this, a continuous global improvement in diagnostic tests remains crucial for a faster diagnosis to treat possible positive patients and implement measures of containment, in both industrialized countries and low-resource settings [18,37]. 

## 5. Conclusions

A “homemade” SYBR^®^Green-based rRT-PCR, designed to detect the N gene of SARS-CoV-2, exhibited a high and reliable method for detection. This suggests the importance of developing front-line and low-cost analytical procedures even during a health emergency. In this case, territorial screening delivered the possibility of an early response between suspected clinical cases and SARS-CoV-2 infection. This possibility has allowed targeted epidemiological choices during the pandemic initial phase of COVID-19 pandemic in Sardinia island. 

### Recommendation

While more research is needed to elucidate the true nature of the severe acute respiratory syndrome, the *Caterina assay* should be used rather than imported commercialized kits because it is a cheaper and reliable way to detect COVID-19.

## Figures and Tables

**Table 1:** Clinical performance comparison between Genesis-coronavirus/DA An Gene Co., Ltd. kit and *Caterina assay* (*n* = 210).

	COVID-19 Genesig Real-Time PCR Assay^TM^			DA An Gene (2019-nCoV) RNA^TM^		
Caterina Assay	Detected	No Detected	Kappa (±95% CI) *	Detected	No Detected	Kappa (±95% CI) *
Detected	110	3	0.971 (0.939–1.0)	112	1	0.990 (0.972–1.0)
No Detected	0	97		0	97	

* Upper/lower (±) 95% CI. *Caterina assay* vs. Genesig**^TM^** Real Time as Gold Standard, sensitivity = 100%, specificity = 97%, positive predictive value = 93.7%, negative predictive value = 100%.

## Data Availability

The raw data supporting the conclusions of this manuscript will be made available by the authors. However, data sharing is not applicable to this article in case of patients data.

## References

[1] Gorbalenya A.E., Baker S.C., Baric R.S., de Groot R.J., Drosten C., Gulyaeva A.A., Haagmans B.L., Lauber C., Leontovich A.M., Neuman B.W. (2020). The species Severe acute respiratory syndrome-related coronavirus: Classifying 2019-nCoV and naming it SARS-CoV-2. Nat. Microbiol..

[2] Carta M.G., Orrù G., Scano A., Coghe F., Nunnari G., Facchini G., Numis F.G., Berretta M. (2020). In the face of the SARS-CoV-2 outbreak, do people suffering from oncological disease need specific attention?. Eur. Rev. Med. Pharmacol. Sci..

[3] Saban O., Levy J., Chowers I. (2020). Risk of SARS-CoV-2 transmission to medical staff and patients from an exposure to a COVID-19-positive ophthalmologist. Graefe’s Arch. Clin. Exp. Ophthalmol..

[4] Ferrando M.L., Coghe F., Scano A., Carta M.G., Orrù G. (2021). Co-infection of Streptococcus pneumoniae in Respiratory Infections Caused by SARS-CoV-2. Biointerface Res. Appl. Chem..

[5] Wiersinga W.J., Rhodes A., Cheng A.C., Peacock S.J., Prescott H.C. (2020). Pathophysiology, Transmission, Diagnosis, and Treatment of Coronavirus Disease 2019 (COVID-19): A Review. JAMA.

[6] Godri Pollitt K.J., Peccia J., Ko A.I., Kaminski N., Dela Cruz C.S., Nebert D.W., Reichardt J.K.V., Thompson D.C., Vasiliou V. (2020). COVID-19 vulnerability: The potential impact of genetic susceptibility and airborne transmission. Hum. Genom..

[7] WHO Weekly Epidemiological Update—29 December 2020. https://www.who.int/publications/m/item/weekly-epidemiological-update.

[8] Lu H., Stratton C.W., Tang Y. (2020). The Wuhan SARS-CoV-2—What’s next for China. J. Med. Virol..

[9] Galli C., Plebani M. (2020). Clinical laboratory and SARS-CoV-2 infection: Where do we stand?. Clin. Chem. Lab. Med..

[10] Mathuria J.P., Yadav R. (2020). Rajkumar Laboratory diagnosis of SARS-CoV-2—A review of current methods. J. Infect. Public Health.

[11] Holland P.M., Abramson R.D., Watson R., Gelfand D.H. (1991). Detection of specific polymerase chain reaction product by utilizing the 5′----3′ exonuclease activity of Thermus aquaticus DNA polymerase. Proc. Natl. Acad. Sci. USA.

[12] (2020). Centers for Disease Control and Prevention CDC 2019-Novel Coronavirus (2019-nCoV) Real-Time RT-PCR Diagnostic Panel. https://www.cdc.gov/coronavirus/2019-ncov/lab/rt-pcr-panel-primer-probes.html.

[13] Corman V.M., Landt O., Kaiser M., Molenkamp R., Meijer A., Chu D.K.W., Bleicker T., Brünink S., Schneider J., Schmidt M.L. (2020). Detection of 2019 novel coronavirus (2019-nCoV) by real-time RT-PCR. Eurosurveillance.

[14] Corman V., Bleicker T., Brunink S., Drosten C. (2020). Diagnostic detection of Wuhan coronavirus 2019 by real-time RT-PCR. Public Health Engl..

[15] Moore N.M., Li H., Schejbal D., Lindsley J., Hayden M.K. (2020). Comparison of Two Commercial Molecular Tests and a Laboratory-Developed Modification of the CDC 2019-nCoV Reverse Transcriptase PCR Assay for the Detection of SARS-CoV-2. J. Clin. Microbiol..

[16] Iglói Z., Leven M., Abdel-Karem Abou-Nouar Z., Weller B., Matheeussen V., Coppens J., Koopmans M., Molenkamp R. (2020). Comparison of commercial realtime reverse transcription PCR assays for the detection of SARS-CoV-2. J. Clin. Virol..

[17] Broughton J.P., Deng X., Yu G., Fasching C.L., Servellita V., Singh J., Miao X., Streithorst J.A., Granados A., Sotomayor-Gonzalez A. (2020). CRISPR–Cas12-based detection of SARS-CoV-2. Nat. Biotechnol..

[18] Carta M.G., Scano A., Lindert J., Bonanno S., Rinaldi L., Fais S., Orrù G. (2020). Association between the spread of COVID-19 and weather-climatic parameters. Eur. Rev. Med. Pharmacol. Sci..

[19] Arcadu B., Orrù M., Piga R., Orrù G. (2012). Designing of sequencing assay assisted by capillary electrophoresis based on DNA folding analysis: An application to the VCAM1 gene. Electrophoresis.

[20] Orrù G., Ferrando M.L., Meloni M., Liciardi M., Savini G., De Santis P. (2006). Rapid detection and quantitation of Bluetongue virus (BTV) using a Molecular Beacon fluorescent probe assay. J. Virol. Methods.

[21] Sumner K., Swensen J.J., Procter M., Jama M., Wooderchak-Donahue W., Lewis T., Fong M., Hubley L., Schwarz M., Ha Y. (2014). Noncontinuously Binding Loop-Out Primers for Avoiding Problematic DNA Sequences in PCR and Sanger Sequencing. J. Mol. Diagn..

[22] WHO Announces COVID-19 Outbreak a Pandemic. https://www.euro.who.int/en/health-topics/health-emergencies/coronavirus-covid-19/news/news/2020/3/who-announces-covid-19-outbreak-a-pandemic.

[23] Petrillo S., Carrà G., Bottino P., Zanotto E., De Santis M.C., Margaria J.P., Giorgio A., Mandili G., Martini M., Cavallo R. (2020). A Novel Multiplex qRT-PCR Assay to Detect SARS-CoV-2 Infection: High Sensitivity and Increased Testing Capacity. Microorganisms.

[24] Zhen W., Berry G.J. (2020). Development of a New Multiplex Real-Time RT-PCR Assay for Severe Acute Respiratory Syndrome Coronavirus 2 (SARS-CoV-2) Detection. J. Mol. Diagn..

[25] Kashir J., Yaqinuddin A. (2020). Loop mediated isothermal amplification (LAMP) assays as a rapid diagnostic for COVID-19. Med. Hypotheses.

[26] Zhang Y.-Z. (2020). Novel 2019 coronavirus genome. Virological. Org..

[27] Li D., Zhang J., Li J. (2020). Primer design for quantitative real-time PCR for the emerging Coronavirus SARS-CoV-2. Theranostics.

[28] Dorlass E.G., Monteiro C.O., Viana A.O., Soares C.P., Machado R.R.G., Thomazelli L.M., Araujo D.B., Leal F.B., Candido E.D., Telezynski B.L. (2020). Lower cost alternatives for molecular diagnosis of COVID-19: Conventional RT-PCR and SYBR Green-based RT-qPCR. Braz. J. Microbiol..

[29] Guglielmotti M.B., Olmedo D.G., Cabrini R.L. (2000). Research on implants and osseointegration. Periodontology.

[30] Chu D.K.W., Pan Y., Cheng S.M.S., Hui K.P.Y., Krishnan P., Liu Y., Ng D.Y.M., Wan C.K.C., Yang P., Wang Q. (2020). Molecular Diagnosis of a Novel Coronavirus (2019-nCoV) Causing an Outbreak of Pneumonia. Clin. Chem..

[31] Wu C., Qavi A.J., Hachim A., Kavian N., Cole A.R., Moyle A.B., Wagner N.D., Sweeney-Gibbons J., Rohrs H.W., Gross M.L. (2020). Characterization of SARS-CoV-2 N protein reveals multiple functional consequences of the C-terminal domain. bioRxiv.

[32] Surjit M., Lal S.K. (2008). The SARS-CoV nucleocapsid protein: A protein with multifarious activities. Infect. Genet. Evol..

[33] Moreira R.A., Guzman H.V., Boopathi S., Baker J.L., Poma A.B. (2020). Characterization of Structural and Energetic Differences between Conformations of the SARS-CoV-2 Spike Protein. Materials.

[34] Vilar S., Isom D.G. (2021). One Year of SARS-CoV-2: How Much Has the Virus Changed?. Biology.

[35] Chen A.T., Altschuler K., Zhan S.H., Chan Y.A., Deverman B.E. (2020). COVID-19 CG: Tracking SARS-CoV-2 mutations by locations and dates of interest. bioRxiv.

[36] CDC, Centers for Disease Control and Emerging SARS-CoV-2 Variants https://www.cdc.gov/coronavirus/2019-ncov/more/science-and-research/scientific-brief-emerging-variants.html.

[37] Carta M.G., Romano F., Orrù G. (2020). The True Challenges of the Covid-19 Epidemics: The Need for Essential Levels of Care for All. Open Respir. Med. J..

